# A Multiple *N*-Glucosylated Peptide Epitope Efficiently Detecting Antibodies in Multiple Sclerosis

**DOI:** 10.3390/brainsci10070453

**Published:** 2020-07-15

**Authors:** Francesca Nuti, Feliciana Real Fernandez, Giuseppina Sabatino, Elisa Peroni, Barbara Mulinacci, Ilaria Paolini, Margherita Di Pisa, Caterina Tiberi, Francesco Lolli, Martina Petruzzo, Roberta Lanzillo, Vincenzo Brescia Morra, Paolo Rovero, Anna Maria Papini

**Affiliations:** 1 Interdepartmental Research Unit of Peptide and Protein Chemistry and Biology, Department of Chemistry “Ugo Schiff”, University of Florence, Via della Lastruccia 13, 50019 Sesto Fiorentino, Italy; francesca.nuti@unifi.it (F.N.); feliciana.realfernandez@unifi.it (F.R.F.); giuseppina.sabatino@cnr.it (G.S.); elisa.peroni@cyu.fr (E.P.); barbaramulinacci@gmail.com (B.M.); ilariapao@gmail.com (I.P.); marghedipi@gmail.com (M.D.P.); caterina.tiberi@gmail.com (C.T.); 2 CNR-IC Istituto di Cristallografia, Via Paolo Gaifami 18, 95126 Catania, Italy; paolo.rovero@unifi.it; 3 UMR 8076 CNRS-BioCIS Team of Chemical Biology and PeptLab@UCP Platform of Peptide and Protein Chemistry and Biology, Neuville Campus, CY Cergy Paris Université, 5 mail Gay-Lussac, 95031 Cergy-Pontoise CEDEX, France; 4 Department of Clinical and Experimental Biomedical Sciences “Mario Serio” and Careggi University Hospital, University of Florence, Largo Brambilla 3, 50134 Florence, Italy; lolli@unifi.it; 5 Multiple Sclerosis Clinical Care and Research Centre, Department of Neurosciences, Reproductive Sciences and Odontostomatology, Federico II University, Via Sergio Pansini 5, 80131 Naples, Italy; martinapetruzzo@gmail.com (M.P.); roberta.lanzillo@unina.it (R.L.); vincenzo.bresciamorra2@unina.it (V.B.M.); 6 Interdepartmental Research Unit of Peptide and Protein Chemistry and Biology, Department of Neurosciences, Psychology, Drug Research and Child Health–Section of Pharmaceutical Sciences and Nutraceutics, University of Florence, Via Ugo Schiff 6, 50019 Sesto Fiorentino, Italy

**Keywords:** Multiple Sclerosis, antibody detection, ELISA, multivalency, N-glucosylated peptide epitopes

## Abstract

Diagnostics of Multiple Sclerosis (MS) are essentially based on the gold standard magnetic resonance imaging. Few alternative simple assays are available to follow up disease activity. Considering that the disease can remain elusive for years, identification of antibodies fluctuating in biological fluids as relevant biomarkers of immune response is a challenge. In previous studies, we reported that anti-*N*-glucosylated (*N*-Glc) peptide antibodies that can be easily detected in Solid-Phase Enzyme-Linked ImmunoSorbent Assays (SP-ELISA) on MS patients’ sera preferentially recognize hyperglucosylated adhesin of non-typeable *Haemophilus Influenzae*. Since multivalency can be useful for diagnostic purposes to allow an efficient coating in ELISA, we report herein the development of a collection of Multiple *N*-glucosylated Peptide Epitopes (*N*-Glc MEPs) to detect anti-*N*-Glc antibodies in MS. To this aim, a series of *N*-Glc peptide antigens to be represented in the *N*-GlcMEPs were tested in competitive ELISA. We confirmed that the epitope recognized by antibodies shall contain at least 5-mer sequences including the fundamental *N*-Glc moiety. Using a 4-branched dendrimeric lysine scaffold, we selected the *N*-Glc MEP **24**, carrying the minimal epitope Asn(Glc) anchored to a polyethylene glycol-based spacer (PEG) containing a 19-atoms chain, as an efficient multivalent probe to reveal specific and high affinity anti-*N*-Glc antibodies in MS.

## 1. Introduction

Multiple Sclerosis (MS) is the most frequent, chronic, inflammatory, demyelinating, disabling disease of the central nervous system, mainly caused by an autoimmune response to self-antigens in genetically susceptible individuals. MS diagnosis and prognosis are mainly supported by magnetic resonance imaging (MRI) that up to now is considered the gold standard diagnostic technique [1]. To the best of our knowledge, there is still no biological marker relevant not only for MS diagnosis but also for its prognosis [2,3,4]. In the last few years, the role of autoantibodies in MS and their identification have been re-evaluated [5,6,7,8]. In particular, a cell-based assay to detect antibodies to myelin oligodendrocyte glycoprotein (MOG), one of the candidate protein autoantigens in MS, has been proposed. However, the real antigen(s) responsible of anti-MOG antibody recognition in the assay remain elusive.

Covid-19, triggering the coronavirus pandemic era we have been living in 2020, marks the return of the old and familiar, but unfortunately misunderstood, enemy killing more human beings than natural disasters: viruses, bacteria, and parasites killers that our modern world tried to fight mainly by social distancing.

Historically, protein antigens isolated from biological material or reproduced by recombinant technologies were used to detect antibodies, but this approach has sometimes turned out as unrealistic. The main limiting factor depends on epitope recognition because of sequence mutations, incorrect folding, lack of post-translational modifications (aberrant versus native), and nonspecific binding.

Peptides mimicking the appropriate epitopes can be valuable tools. In fact, peptides that can be synthetically produced as unique molecules, can increase specificity of antibody recognition, eliminating or minimizing potential cross-reactivity with structurally similar fragments in non-relevant proteins [9,10,11].

Several Enzyme-Linked ImmunoSorbent Assays (ELISA) based on synthetic peptides have been proposed to detect antibodies in different diseases like Acquired ImmunoDeficiency Syndrome (AIDS), Infectious Bronchitis (IB), Severe Acute Respiratory Syndrome (SARS), and Bluetongue (BT) [12,13]. However, peptide-based ELISA can have some practical limitations, such as the antigen immobilization that can ultimately affects the sensitivity of the assay, particularly when short sequences (7–8 amino acids) are used [14,15]. Surface functionalization and biomolecular interactions can overcome these disadvantages, such as in the streptavidin-biotin system [16].

An interesting strategy to increase surface binding on the ELISA plate, improving sensitivity, is based on multimeric peptide dendrimers. In particular, Multiple Antigen Peptides (MAPs) are an optimal compromise between short peptide epitopes and recombinant or native antigens. It is widely recognized, that surface antibody binding is increased by multivalent presentation of the antigen. In fact, multivalent interactions can be collectively much stronger than the sum of the corresponding monovalent interactions [17,18]. Therefore, MAPs represent a useful chemically unambiguous system to explore antigen-antibody interaction, thanks to their shape and globular physical characteristics [19,20].

Moreover, aberrant Post-Translational Modifications (PTMs) of antigens, can play a fundamental role in triggering antibodies. Particularly, the *N*-glycosylation has been described as possible PTM involved in an antibody-mediated form of MS [21,22]. 

In previous studies, by a structure-based design, we developed a collection of synthetic glycopeptides, characterized by β-turn structures optimally exposing the sugars as minimal epitopes. We demonstrated that the β-d-glucopyranosyl moiety (Glc) linked to an Asn residue (*N*-Glc) on the tip of the turn is fundamental for antibody recognition in an MS patients’ population. *N*-Glc is a prokaryote-specific modification that is found in selected Gram-negative bacteria, where it is most commonly found on cell-surface proteins such as (autotransporter) adhesins, biosynthesized as part of the three-protein HMW cluster including the *N*-glucosyl transferase HMW1C. We demonstrated that anti-*N*-Glc peptide antibodies, easily detected by a Solid-Phase (SP)-ELISA [22,23], preferentially recognize hyperglucosylated adhesin of non-typeable *Haemophilus influenzae* (NTHi) particularly the C-terminal portion HMW1(1205-1526) termed HMW1ct. This was the first example of an N-glucosylated native antigen that can be considered a relevant candidate for triggering pathogenic antibodies in MS [24]. The protein HMW1ct is expressed as a mixture of three *N*-Glc variants containing 7, 8, and 9 Glc moieties on Asn residues inside the consensus sequence NX(S/T) in a 1:1:1 ratio. Since the NTHi cell-surface adhesins are widely glucosylated, the *N*-Glc residues are likely to be exposed conceptually in vivo in a multivalent shape, thus potentially favoring the emergence of a rough immunological response.

In the present study, we aimed to reproduce multivalent exposure of *N*-Glc epitopes to increase coating efficiency on the ELISA microplate for the detection of anti-*N*-Glc antibodies in MS, reminiscent of an early infection. First of all, by ELISA experiments, both competitive and in solid-phase, we defined the *N*-Glc epitopes (assuring the specificity and selectivity of autoantibody recognition), decreasing the length of the originally developed *N*-glucosylated β-turn synthetic antigenic probes. Then, the best efficiency of coating to the polystyrene ELISA plate was guaranteed, considering the concept of the multivalency to increase the antibody binding affinity. In particular, the selected short epitopes were conjugated to 4-branched dendrimeric lysine scaffolds creating Multiple *N*-Glucosylated Peptide Epitopes (*N*-Glc MEPs). Therefore, the novel *N*-Glc MEPs were developed with the aim not only to enhance the diagnostic performance of the assay but also the coating efficiency of the minimal epitopes to the polystyrene ELISA plate.

## 2. Materials and Methods

### 2.1. Synthesis of the N-Glc Peptides

*N*-Glc peptide epitopes **2–22** (Table 1 and Table 2) were synthesized following the Fmoc/tBu manual strategy. Experimental details and analytical data of the synthetic molecules are reported in the Supplementary Materials (Tables S1 and S2).

### 2.2. Synthesis of N-Glc Multiple Epitope Peptides (N-Glc MEPs)

*N*-Glc MEPs **23**–**26** (see Section 3.4) were synthesized following the protocol described in the Supplementary Materials. Analytical data are reported in Table S3.

### 2.3. Immunological Assays 

Multiple Sclerosis (MS) patients’ sera samples were collected in the Multiple Sclerosis Clinical Care and Research Centre, Department of Neurosciences, Reproductive Sciences and Odontostomatology, Federico II University (Naples, Italy). Sera samples were obtained for diagnostic purposes, from patients and healthy blood donors who had given their informed consent, and stored at −20 °C until use. The present study was conducted in accordance with the Declaration of Helsinki. All experimental protocols performed were approved by the Ethics Committee 2006 and 2017 (protocol n. 120/06 and 160/17, respectively). The MS group consisted of relapsing-remitting MS (RR-MS) patients after a diagnostic lumbar puncture, cerebrospinal fluid analysis, and MRI examination and fulfilled established international diagnostic criteria [25,26]. Blood samplings in the patients’ group were performed during the routine follow-up study, while the healthy control samples were carried out during routine health checks or blood donations.

#### 2.3.1. Inhibition ELISA

Ninety-six-well activated polystyrene ELISA plates (NUNC Maxisorb, Sigma Aldrich, Milano, Italy) were coated with 1 μg per 100 μL of the type I’ beta turn glucosylated peptide CSF114(Glc) or MEPs per well, in pure carbonate buffer 0.05 M (pH 9.6) and incubated at 4 °C overnight. Washing steps were executed with an automatic Hydroflex microplate washer (Tecan Italia, Milano, Italy). After five washes with washing buffer containing 0.9% NaCl and 0.05% Tween 20, nonspecific binding sites were blocked with 100 μL per well fetal calf serum (FCS) buffer solution (10% in washing buffer) at room temperature for 60 min. 

Antibody affinity was measured following the inhibition methods reported elsewhere [27,28]. Semi-saturating sera dilution was calculated in preliminary titration curves (absorbance 0.7). Six different concentrations of each synthetic antigenic peptide probe were used as inhibitors. Then, sera samples at the selected dilution were incubated in parallel with increasing concentrations of the synthetic shortened peptide sequences (range 1 × 10^−10^ to 1 × 10^−4^) for 60 min at room temperature. All inhibition experiments were performed in duplicate or triplicate for each single MS patient’s serum positive to CSF114(Glc) separately. All experiments were repeated at least twice on two different working days.

After three washes, uninhibited antibodies were identified by adding 100 µL/well of alkaline phosphatase-conjugated anti-human immunoglobulin G (IgG, Sigma-Aldrich, Milano, Italy) diluted 1:8000 in washing buffer containing 10% FCS. The microplates were then incubated 3 h at room temperature and, after three washes, 100 µL of substrate solution consisting of 1 mg/mL *p*-nitrophenyl phosphate (Sigma-Aldrich, Milano, Italy) in 10% diethanolamine buffer (pH 9.8) were added. After approximately 30 min, the reaction was stopped with 1M NaOH solution (50 µL/well), and the absorbance was read in a multichannel ELISA reader (Tecan Sunrise, Männedorf, Switzerland) at 405 nm. The selected ELISA microplates, coating conditions, reagent dilutions, buffers, and incubation times were previously tested [24,29]. The relationship between peptide concentrations and the absorbance values was represented graphically in absorbance inhibition percentage, and half-maximal response concentration values (IC50) were calculated.

#### 2.3.2. Solid-Phase ELISA (SP-ELISA) 

Immunoassays, to detect IgM or IgG antibodies in sera, were performed by SP-ELISA coating the synthetic peptides on 96-well plates (Nunc Maxisorp, Sigma−Aldrich, Milano, Italy). 

Polystyrene 96-well ELISA plates were coated with a 10 μg/mL solution of diluted synthetic peptides, independently, in pure carbonate buffer 0.05 M (pH 9.6). After overnight incubation at 4 °C, plates were washed three times using washing buffer. Nonspecific binding sites were blocked with 100 μL/well of fetal calf serum buffer (10% FCS in washing buffer) at room temperature for 1 h. FCS buffer was removed and plates were incubated overnight with sera (diluted 1:100 in FCS buffer, 100 μL/well) at 4 °C. After three washes, plates were treated with 100 μL/well of anti-human IgG or IgM alkaline phosphatase-conjugated specific antibodies diluted in FBS buffer (1:8000 and 1:200, respectively). After 3 h of incubation at room temperature and three washes, 100 μL of substrate buffer was added to each well. After 15–30 min incubation at room temperature, the absorbance of each plate was read in a multichannel ELISA reader at 405 nm. The antibody levels are expressed as absorbance in arbitrary units at 405 nm.

### 2.4. Statistical Analysis 

Data are expressed as measured absorbance values at 405 nm calculated as the mean ± SD. Statistical analysis was performed using the software GraphPad Prism version 6.01 (Graphpad Software Inc., La Jolla, CA, USA). Descriptive statistics was used to calculate mean and standard deviation for continuous variables and percentage for categorical variables. Mann–Whitney U tests were used to compare antibody response distributions. Differences were deemed statistically significant when *p* value < 0.05 (two-tailed test). Non-parametric Spearman’s rho and related 95% confidence intervals were used to assess correlation between pair of tests. A *p* value < 0.05 (two-tailed test) was considered as significant. Receiver Operating Characteristic (ROC) curve analysis was employed to calculate cut-off values, establishing sensitivities and sensibilities.

## 3. Results

In previous studies, we reported the cross-reactivity between the *N*-glucosylated adhesin antigen HMW1ct-Glc and anti-CSF114(*N*-Glc) IgG antibodies in MS patients’ sera by competitive ELISA [25]. The HMW1C from NTHi is one of the first examples of soluble bacterial protein glycosyltransferases capable of performing *N*-glycosylation with simple hexoses (i.e., glucose) on asparagine residues in conserved Asn-Xaa-Ser/Thr motifs [29,30]. On the other hand, CSF114(Glc) is a structure-based designed 21-mer peptide (TPRVERN(Glc)GHSVFLAPYGWMVK) that was optimized as a type I’ beta-turn structure because of its ability to expose at the best, at position 7, the minimal, but fundamental moiety Asn(*N*-Glc), in the epitope for autoantibody recognition in the solid-phase conditions of the immunoenzymatic assay [31].

Starting from this assumption, preliminary experiments were performed to select the shortest peptide sequences corresponding to the epitope(s) to be presented in multiple copies in fully characterized multivalent Multiple Epitope Peptides (MEPs) to detect antibodies in MS patients’ sera by SP-ELISA. The use of MEPs for specific antibody detection can pave the way for the development of a simple tool to identify immune responses to aberrant glycosylations, such as *N*-glucosylation in MS possibly linked to an early non-typeable *Haemophilus influenzae* bacterial infection.

### 3.1. Antibody Detection in Solid-Phase ELISA (SP-ELISA) Is Affected by the Length of the Peptide Antigen 

First of all, we investigated the influence of the length of shortened peptide sequences of the synthetic antigenic probe CSF114(Glc) on the efficiency both in antibody recognition and on the coating in the SP-ELISA. At this purpose, the glucopeptide sequence was tightened down step-by-step and the shortened peptides **2–5** (Table 1) derived from CSF114(Glc) were synthesized (as described in the Supplementary Materials) and characterized using analytical Reverse-Phase High Performance Liquid Chromatography (RP-HPLC) and ElectroSpray Ionisation Mass Spectrometry (ESI-MS) (Table S1). The shortened peptide sequences were acetylated at the *N*-terminus and amides at the *C*-terminus, in order to dislodge free terminal charges, which are not present in the native protein sequence and might hamper the antibody recognition [32]. 

**Figure 1:**
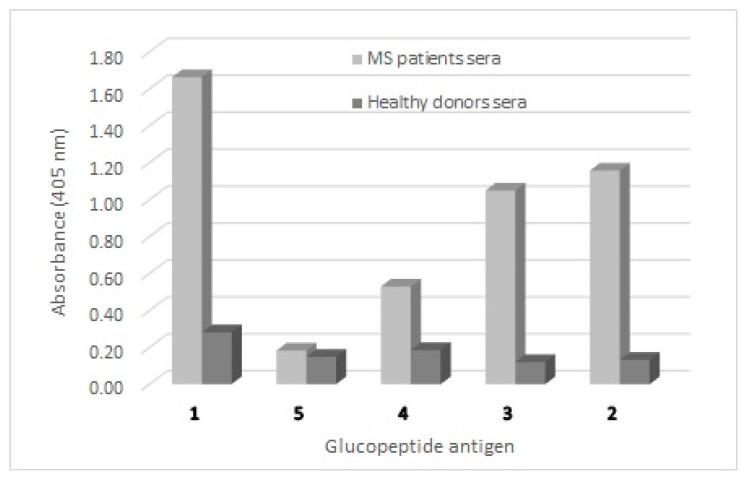
Antibody titres (expressed as Absorbance values) in Multiple Sclerosis (MS) patients’ and healthy blood donors’ sera against the glucopeptide antigens CSF114(Glc) (**1**), [Asn^7^(Glc)]CSF114(1–18) (**2**), [Asn^7^(Glc)]CSF114(1–16) (**3**), [Asn^7^(Glc)]CSF114(1–14) (**4**), and [Asn^6^(Glc)]CSF114(2–13) (**5**).

SP-ELISA, against pools of positive MS sera and healthy controls (Figure 1), clearly showed a progressive decrease in antibody titre in parallel with the decrease in peptide length. In fact, the 12-mer glucopeptide **5** completely lost its ability to identify antibodies in SP-ELISA, possibly because of an inefficient coating. Therefore 14 residues appeared to be the minimum length necessary for antibody identification in SP-ELISA, probably because shorter immobilized glucopeptide sequences (<14) are only partially prone to expose the epitope for antibody binding.

### 3.2. Antibody Affinity of Shortened CSF114(Glc) Glucopeptide Sequences in Competitive ELISA: Shortening CSF114(Glc) Does not Affect Antibody Epitope Recognition

Considering that SP-ELISA allows to evaluate fundamentally the relative antibody affinity that depends on the exposure of the peptide in the solid-phase conditions of the assay, we also investigated the absolute antibody affinity against the CSF114(Glc) shortened sequences by a competitive ELISA. For this purpose, the *N*-glucosylated β-turn peptide structure CSF114(Glc) was tightened down again step-by-step with the aim to identify the critical epitope displaying optimal antibody binding reactivity in the competitive in vitro assay. 

Further, we investigated the role of the amino acids surrounding the previously identified minimal epitope Asn(*N*-Glc) [24]. In particular, we developed the synthetic shorter sequences **6–8** that were tested in competitive ELISA in parallel to **2–5** (Table 1).

**Figure 2:**
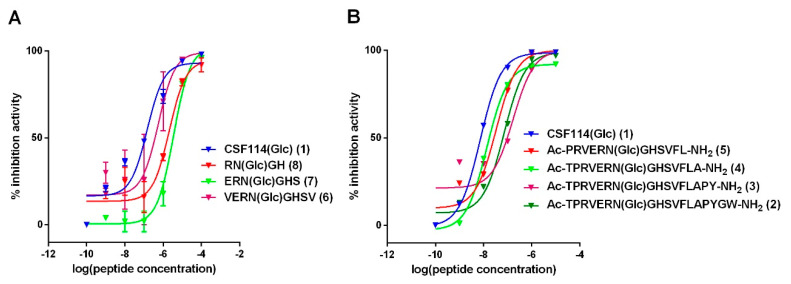
Inhibition curves: (**A**) Inhibition curve of anti-CSF114(Glc) IgG antibodies from a representative MS serum with *N*-glucosylated peptides, [Asn^7^(Glc)]CSF114(4–11) (**6**), [Asn^7^(Glc)]CSF114(5–10) (**7**), and [Asn^7^(Glc)]CSF114(6–9) (**8**) in comparison with the *N*-glucosylated peptide CSF114(Glc) (**1**) in a competitive Enzyme-Linked ImmunoSorbent Assay (ELISA). (**B**) Inhibition curve of anti-CSF114(Glc) IgG antibodies from a representative MS serum with glucopeptides: [Asn^7^(Glc)]CSF114 (1–18) (**2**), [Asn^7^(Glc)]CSF114 (1–16) (**3**), [Asn^7^(Glc)]CSF114 (1–14) (**4**), [Asn^7^(Glc)]CSF114 (2–13) (**5**) in comparison with the glucopeptide CSF114(Glc) in a competitive ELISA. The results are expressed as percentage of inhibition activity of representative MS serum (ordinate axis) versus the peptide concentrations (M) in logarithmical scale.

All the synthetic glucopeptides were used as inhibitors in competitive ELISA using anti-CSF114(Glc) antibodies in one representative positive MS patient’s serum. The IC50 s were calculated applying the non-linear regression least squares (ordinary) fit to the experimental data for each peptide, and values are summarized in the Supplementary Materials (Table S4). As shown in Figure 2, the full length CSF114(Glc) showed the highest degree of binding affinity (IC50 = 0.009 µM, Table S4 in the Supplementary Materials). Despite 14 residues are the minimum for antibody recognition in SP-ELISA, the inhibitory activity is still optimal when the epitope region is included in ca. 11 amino acid residues as **5** and **4** display (IC50 = 0.035 µM and 0.014 µM, respectively). On the other hand, the 4-mer glucopeptide **8** and the 6-mer glucopeptide **7** displayed the lowest inhibitory potency (IC50 = 2.2 µM and 3.5 µM, respectively), but, in any case, all are able to inhibit antibodies.

### 3.3. Study of the Possible Role of a Consensus Sequence Surrounding N(Glc) in Antibody Recognition

Taking into consideration that shorter glucopeptides **6** and **8** displayed antibody affinity in competitive ELISA, we decided to investigate a series of *N*-Glc tri- and pentapeptides as synthetic antigens, undertaking a deductive approach to rule out *N*-glycosylation consensus sequences (sequons).

As a proof of concept, among the possible combinations of tripeptide sequons NX(T/S), we selected the ones containing X = Gly or Lys. In particular, we synthesized *N*(Glc)GS (**9**), *N*(Glc)GT (**10**), *N*(Glc)KS (**11**), and *N*(Glc)KT (**12**). Moreover, we synthesized *N*(Glc)GH (**13**) and *N*(Glc)KH (**14**), derived from the original glucosylated core of CSF114(Glc), that did not correspond to a consensus sequence. Then, we included the sequon *N*(Glc)AT (**15**), present both in the human myelin oligodendrocyte glycoprotein (MOG) sequence [Asn^31^(Glc)]hMOG(30–50) (an antigenic immunodominant epitope recognizing also antibodies in MS) [33], but also highly represented (3 out of 12 positions) in the C-terminal portion HMW1(1205–1526), i.e., HMW1ct hyperglucosylated adhesin of non-typeable *Haemophilus influenzae* (NTHi) (N at positions 3, 7, and 9) [25] together with *N*(Glc)GS (**9**). Moreover, considering that a tripeptide sequence is probably not long enough for a good antigen-antibody interaction, the following step in the identification of the epitope was to test the pentapeptides **16–22**, including the sequons. At this purpose the two amino acids placed in positions –B2 and –B1 in the CSF114(Glc) sequence were added to allow the spatial hint in the shape of a β-turn, with Asn(Glc) on the tip. The same idea was applied to the sequon *N*(Glc)AT (**15**), inserting amino acids in positions 29 and 30 of hMOG(30–50) sequence, originally containing Asn(Glc) at position 31 [33].

Therefore, glucosylated tri- and pentapeptides **9–22** (Table 2) were synthesized, purified, and characterized as described in detail in the Supplementary Materials. 

**Figure 3:**
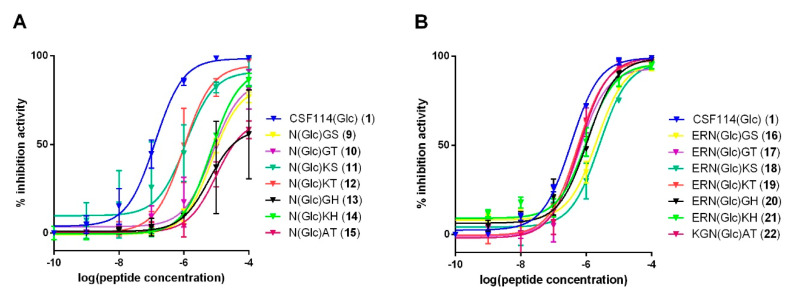
Inhibition curves: (**A**) Inhibition curve of anti-CSF114(Glc) IgG antibodies from a representative MS serum with the tripeptides **9–15**: Ac-N(Glc)GS-NH_2_ (**9**), Ac-N(Glc)GT-NH_2_ (**10**), Ac-N(Glc)KS-NH_2_ (**11**), Ac-N(Glc)KT-NH_2_ (**12**), Ac-N(Glc)GH-NH_2_ (**13**) Ac-N(Glc)KH-NH_2_ (**14**), Ac-N(Glc)AT-NH_2_ (**15**), in comparison with the glucopeptide CSF114(Glc) (**1**) in a competitive ELISA. (**B**) Inhibition curve of anti-CSF114(Glc) IgG antibodies from a representative MS serum with the pentapeptides **16–22**: Ac-ERN(Glc)GS-NH_2_ (**16**), Ac-ERN(Glc)GT-NH_2_ (**17**), Ac-ERN(Glc)KS-NH_2_ (**18**); Ac-ERN(Glc)KT-NH_2_ (**19**), Ac-ERN(Glc)GH-NH_2_ (**20**), Ac-ERN(Glc)KH-NH_2_ (**21**); Ac-N(Glc)AT-NH_2_ (**22**), in comparison with the glucopeptide CSF114(Glc) in a competitive ELISA. The results are expressed as the percentage of inhibition activity of a representative MS serum (ordinate axis) versus the peptide concentrations (M) in logarithmical scale.

The antibody affinity of tripeptides **9–15** and pentapeptides **16–22** was evaluated by competitive ELISA compared to the original *N*-glucosylated β-turn peptide sequence (Figure 3). CSF114(Glc) was coated onto the ELISA microplate and patients’ sera were incubated with different shortened peptide concentrations. IC50 values were reported in the Supplementary Materials (Table S5).

Inhibition curves of the tripeptides **9–15** and pentapeptides **16–22** are shown in Figure 3. The original sequence of CSF114(Glc) presented the best inhibitory activity (IC50 = 0.34 µM, Table S5). The shortest tripeptide probes **9–15** recognized anti-CSF114(Glc) antibodies even if with an affinity lower than the synthetic glucosylated 21mer-peptide (Figure 1). Among all the tripeptides tested, the glucopeptides Ac-N(Glc)KS-NH_2_ (**11**) and Ac-N(Glc)KT-NH_2_ (**12**) showed the best inhibitory activity (IC50 = 1.1 and 0.97 µM, respectively, Table S5).

The longer linear sequences **16–22** exhibited high inhibition efficacy in competitive ELISA, better than their corresponding tripeptide analogs **9–15**. In particular, the pentapeptide Ac-ERN(Glc)KT-NH_2_ (**19**) presented the best inhibitory activity (IC50 = 0.54 µM, Table 3) followed by the pentapeptides Ac-ERN(Glc)GT-NH_2_ (**17**) (IC50 = 0.58 µM) and Ac-KGN(Glc)AT-NH_2_ (**22**) (IC50 = 0.60 µM). This result indicates that the IC50 values for *N*-Glc pentapeptides are closely similar, and no substantial differences were observed among the pentapeptides tested, independently of the sequence.

### 3.4. N-Glc Multiple Epitope Peptides (N-Glc MEPs) to Mimic Multivalency in SP-ELISA

In order to mimic a multivalent presentation of the minimal but fundamental epitope Asn(*N*-Glc), and increasing at the same time the efficiency in coating to the ELISA polystyrene plate of short linear peptides, we synthesized different *N*-Glc MEPs based on a dendrimeric lysine scaffold, having in common Asn(Glc) [34]. 

**Figure 4:**
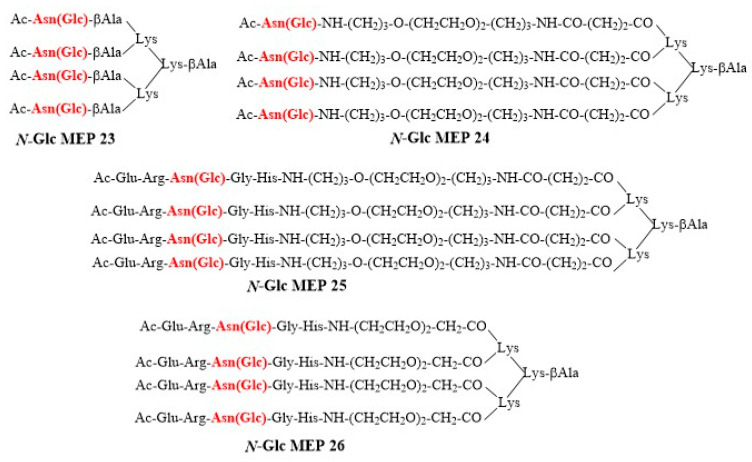
Collection of the synthetic *N*-Glc Multiple Epitope Peptides (MEPs).

Starting from a lysine tetrameric core, we synthesized five *N*-Glc MEPs **23–26** bearing spacers of different length: β-Alanine and two different polyethylene glycol-based spacers (PEG), containing 9- and 19-atoms chain, respectively. The following antigens were selected: (i) the β-turn *N*-glucosylated pentapeptide ERN(Glc)GH (**12**) and (ii) the simple minimal *N*-glucosylated asparagine epitope Asn(Glc). The spacers were introduced to avoid steric hindrance possibly hampering antibody binding. The *N*-Glc MEP structures are reported in Figure 4.

The *N*-Glc MEPs were synthesized starting from Fmoc_4_-Lys_2_-Lys-β-Ala-Wang resin, according to the general procedure described in details in the Supplementary Materials. The Fmoc-Asn(βGlcAc_4_)-OH building block was synthesized according to the method developed by Paolini et al. [35]. *N*-Glc MEPs were acetylated on the *N*-terminal function. All the *N*-Glc MEPs were purified by semi-preparative RP-HPLC, and characterized by RP-HPLC and ESI-MS (Table S3).

**Figure 5:**
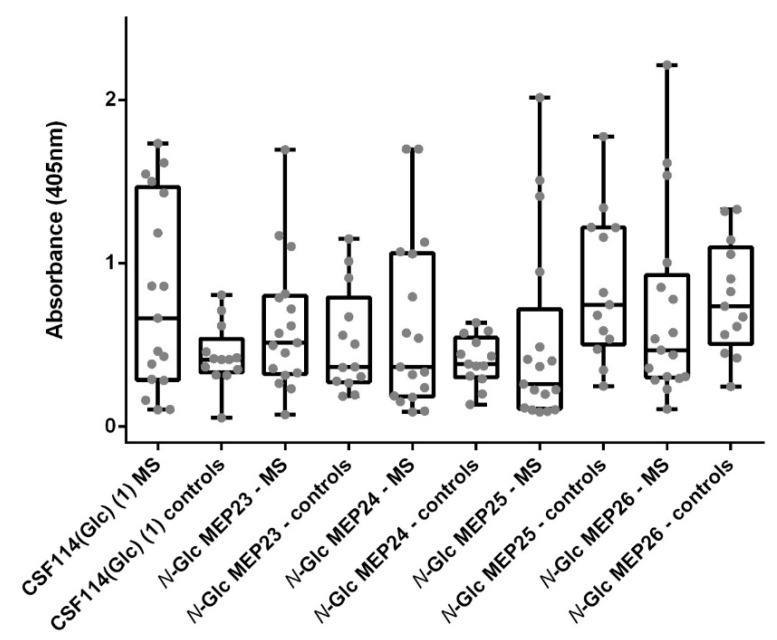
Antibody responses (expressed as Absorbance values) against the synthetic *N*-Glc MEPs. Data distribution of IgG antibodies against the glucopeptide CSF114(Glc) and the synthetic *N*-Glc MEPs **23**–**26** obtained by SP-ELISA (grey). Box and whiskers (min to max) are plotted for each data group (black).

We assessed IgG isotype antibodies against *N*-Glc MEPs **23–26** in a cohort of 16 MS patients, previously selected on their reactivity to the original *N*-glucosylated type I’ β-turn peptide structure CSF114(Glc), and compared to 14 healthy blood donors as controls (Figure 5).

The Receiver Operating Characteristic (ROC) analysis was employed to perform an accurate comparative investigation of the performances of the different *N*-Glc MEPs, evaluating their discrimination power at the different cut-off values. Sensitivity, specificity, and likelihood ratios were also calculated [36]. Selected cut-off values, sensitivities, and the rest of the statistical parameters are shown in Table 3 (ROC curves are reported in Figure S1 in the Supplementary Materials). The characteristics of the curves, their shape and steepness, and their underlying area provide evidence that the synthetic *N*-Glc MEPs are able to identify antibodies in MS patients’ sera. 

Despite *N*-Glc MEP **25** and **26** presented the best Area Under the Curve (AUC) values (0.7511 and 0.6380, respectively), observing the data distribution we assume that their ability to identify specific MS antibodies is lower compared with *N*-Glc MEPs **23** and **24**. This is justified with the increased means of the antibody titers in controls compared to MS patients in both *N*-Glc MEP **25** and **26**, which apparently improved AUC and p values, but are due to non-specific interactions. In fact, the specificities calculated for *N*-Glc MEPs **25** and **26** are sensibly decreased compared with CSF114(Glc) (82.35% and 92.31%, respectively). Considering the sensitivity values, once the specificity is fixed in 92.31%, the *N*-Glc MEP **24** presented the best performance identifying as positive the 35.29% of the MS patients.

**Figure 6:**
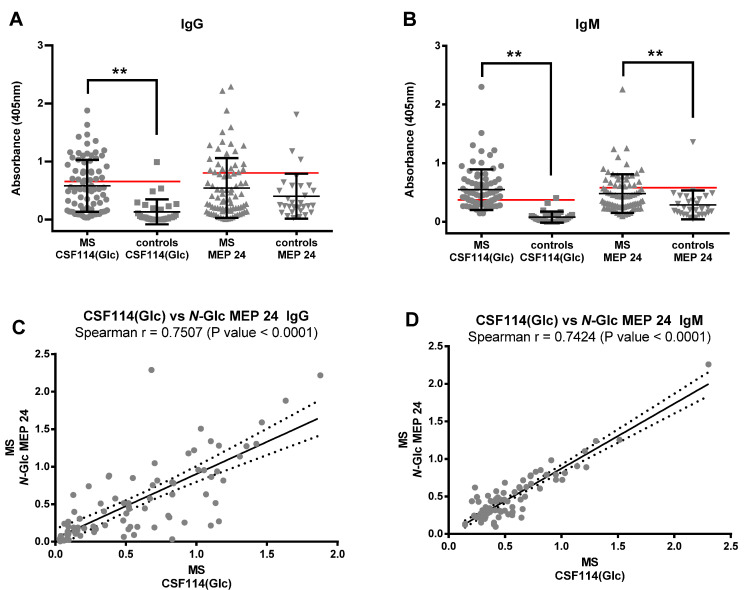
Data of antibody responses against the glucopeptide CSF114(Glc) and *N*-Glc MEP **24**. Data distribution of IgG (**A**) and IgM (**B**) antibody titers in 81 MS patients’ sera and 30 controls’ sera identified by SP-ELISA against the peptide sequences CSF114(Glc) and the synthetic multivalent *N*-Glc MEP **24**. Data are expressed as mean values ± standard deviation with a significance level ** *p* < 0.001 (two-tailed Mann–Whitney non-parametric test). Selected cut-off values for each compound are plotted in red. Correlation between CSF114(Glc) and *N*-Glc MEP **24** both for IgG (**C**) and for IgM (**D**) are shown. The Spearman’s correlation coefficients (rho values) and the corresponding *p* values are reported in each plot. Regressions lines are plotted in black (dashed lines show the 95% confidence interval of the best-fit line).

Consequently, we decided to deepen on *N*-Glc MEP **24** ability (containing only *N*-Glc moieties) to recognize specific MS antibodies in patients’ sera. At this purpose, *N*-Glc MEP **24** was tested against an increased number of MS patients’ sera (81) and healthy controls’ (30) sera, in parallel, to the glucopeptide CSF114(Glc) as reference. Data distribution of IgG and IgM antibody titers identified by SP-ELISA are reported in Figure 6A,B.

The non-parametric Mann–Whitney test was applied to evaluate significant differences between MS patients’ and controls’ groups. The results showed significant differences (*p* value < 0.001, two-tailed test) for CSF114(Glc), both for IgM and IgG-type antibodies. Slightly different results were observed in the case of *N*-Glc MEP **24**. In fact, the differences among groups were statistically significant when IgM-isotype antibodies were detected (*p* value = 0.0001, two-tailed test). On the other hand, the IgGs against the multivalent *N*-Glc MEP **24** showed no level of significance (*p* value = 0.3611, Two-tailed test). In our opinion, the IgG antibody response as detected by *N*-Glc MEP **24** appears to be less specific because it detects a “noise” level in the control group, forcing a decreased specificity to maintain sensibility when selecting the corresponding cut-off.

Among the absorbance values of MS patients, the frequencies of the anti-CSF114(Glc) antibodies significantly correlated with the ones against the *N*-Glc MEP **24** (*p* value < 0.0001, two-tailored); the Spearman’s correlation coefficients (rho values) were *r* = 0.7507 and 0.7424 for IgG and IgM, respectively (Figure 6C,D).

Then, we investigated the absolute antibody affinity of *N*-Glc MEP **24** in a competitive ELISA. In a set of three MS positive sera tested in parallel, the multivalent *N*-Glc MEP **24** inhibited the binding of antibodies to the glycopeptide CSF114(Glc), giving rise to contrasting inhibition curves among the different representative sera employed (Figure S2 in the Supplementary Materials). Data of serum MS1 (Figure S2A in the Supplementary Materials) showed that the affinity of *N*-Glc MEP **24** was lower than CSF114(Glc) (IC50 = 2.145 × 10^−8^ M and 5.200 × 10^−7^ M, respectively), whereas serum MS2 exhibited superimposable affinity (IC50 = 6.373 × 10^−8^ M and 6.088 × 10^−8^ M respectively). Moreover, in MS3 serum IC50 was lower for *N*-Glc MEP **24** compared to CSF114(Glc) (IC50 = 2.145 × 10^−8^ M and 5.116 × 10^−9^ M, respectively). This finding indicates that the *N*-Glc MEP **24** shares similar epitopes, all including the Asn(*N*-Glc) residue. In particular, its antibody affinity can be slightly different among the MS patients, probably because of the differential innate and adaptive immune responses typical of each subject.

## 4. Discussion

Multiple sclerosis diagnosis is still very challenging, relying on clinical and radiological criteria and in the absence of “better explanations” [37], the development of simple diagnostics detecting specific biomarkers is highly warranted. Moreover, native structures triggering specific antibodies in Multiple Sclerosis (MS) are still uncharacterized. Consequently, surrogate antigens used to identify antibodies in MS by ELISA are elusive. In spite of the fact that MS is considered mainly a T-cell mediated disease, the role of B-cells is increasingly appreciated. In this scenario, we demonstrated for the first time that an aberrant *N*-glucosylation is part of a relevant epitope that was identified by the structure-based designed β-turn 21-mer glucopeptide CSF114(Glc). This synthetic tool was instrumental for the discovery of antibodies in an MS patients’ population preferentially recognizing the hyperglucosylated bacterial adhesin of non-typeable *Haemophilus influenzae*. With the idea in mind that multivalent presentation of glucosylated asparagine residues may occur in a variety of native antigens, as in the case of citrullination in rheumatoid arthritis [38], we focused on the development of a synthetic tool enhancing the role of multiple aberrant modifications versus amino acid sequences. We simplified the antigen to be synthetically produced in a multiple format, taking into consideration that peptide dendrimers are considered protein-like multivalent materials, whose architecture is a key parameter for activity [39,40]. Therefore, we selected the multivalent epitope peptide *N*-Glc MEP **24**, based on a lysine-dendritic scaffold (relatively simple to be produced), carrying four copies of the minimal glucosylated epitope Asn(Glc) anchored to a PEG-based spacer containing a 19-atoms chain [41]. In previous studies, we demonstrated that CSF114(Glc) shortened epitopes lost specificity and sensitivity for IgM antibodies in MS patients’ sera by SP-ELISA [29], sensibly decreasing the diagnostic potential of the synthetic antigen. On the contrary, *N*-Glc MEP **24** displays an interesting ability to identify in SP-ELISA both IgG and IgM antibodies in a large number of MS patients’ sera (81) compared to controls (30), with a sensitivity of 35% (95% CI: 14.21–61.67) and a specificity of 92.31 (95% CI: 63.97–99.81). This result is particularly relevant, since it offers the possibility to detect with the same tool both IgMs and IgGs in MS, thus increasing the diagnostic and prognostic value of the antigen thanks to multivalent presentation of the minimal epitope [24].

## 5. Conclusions

The present study reported the development of new *N*-glucosylated Multivalent Epitope Peptides for the detection of anti-*N*(Glc) antibodies in Multiple Sclerosis. After a preliminary screening of several short linear peptide epitopes and multiple epitope peptides, the promising results suggest that multivalent interaction can be useful in designing SP-ELISA to detect antibodies to aberrant *N*-glucosylation in Multiple Sclerosis for both diagnostic and prognostic purposes. Indeed, the multivalent *N*-Glc MEP **24** ligand, carrying the minimal glucosylated epitope Asn (Glc) anchored to the 19-atoms PEG-based spacer, can be an efficient probe to reveal both IgM and IgG autoantibodies as disease biomarkers.

## Figures and Tables

**Table 1:** Shortened *N*-glucosylated (*N*-Glc) sequences of CSF114(Glc).

Peptide Name Number	*N*-Glucosylated Peptide Sequence
CSF114(Glc) (**1**)	TPRVERN(Glc)GHSVFLAPYGWMVK
[Asn^7^(Glc)] CSF114(1–18) (**2**)	Ac-TPRVERN(Glc)GHSVFLAPYGW-NH_2_
[Asn^7^(Glc)] CSF114(1–16) (**3**)	Ac-TPRVERN(Glc)GHSVFLAPY-NH_2_
[Asn^7^(Glc)] CSF114(1–14) (**4**)	Ac-TPRVERN(Glc)GHSVFLA-NH_2_
[Asn^7^(Glc)] CSF114(2–13) (**5**)	Ac-PRVERN(Glc)GHSVFL-NH_2_
[Asn^7^(Glc)] CSF114(4–11) (**6**)	Ac-VERN(Glc)GHSV-NH_2_
[Asn^7^(Glc)] CSF114(5–10) (**7**)	Ac-ERN(Glc)GHS-NH_2_
[Asn^7^(Glc)] CSF114(6–9) (**8**)	Ac-RN(Glc)GH-NH_2_

**Table 2:** *N*-Glc tri- and pentapeptides as synthetic antigens.

Glucosylated Core Peptide	Glucosylated Tripeptides	Glucosylated Pentapeptides
Selected NXT/S sequences	Ac-N(Glc)GS-NH_2_ (**9**); Ac-N(Glc)GT-NH_2_ (**10**); Ac-N(Glc)KS-NH_2_ (**11**); Ac-N(Glc)KT-NH_2_ (**12**).	Ac-ERN(Glc)GS-NH_2_ (**16**); Ac-ERN(Glc)GT-NH_2_ (**17**); Ac-ERN(Glc)KS-NH_2_ (**18**); Ac-ERN(Glc)KT-NH_2_ (**19**)
CSF114(Glc)	Ac-N(Glc)GH-NH_2_ (**13**); Ac-N(Glc)KH-NH_2_ (**14**)	Ac-ERN(Glc)GH-NH_2_ (**20**); Ac-ERN(Glc)KH-NH_2_ (**21**)
[Asn^31^(Glc)]hMOG(30–50)	Ac-N(Glc)AT-NH_2_ (**15**)	Ac-KGN(Glc)AT-NH_2_ (**22**)

**Table 3:** Receiver Operating Characteristic (ROC) analysis. Values obtained from ROC-analysis of *N*-Glc MEPs **23–26** and the glycopeptide CSF114(Glc) (**1**) for the area under the curve, *p* value, established cut-off and the corresponding sensitivity, specificity, and likelihood ratio.

Compound	Area Under Curve (AUC)				Criterion Value and Coordinates			
	AUC	Standard Error (SE)	95% Confidence Interval	*p*-Value	Cut-Off	Sensitivity (%)	Specificity (%)	Likelihood Ratio
*N*-Glc MEP (**23**)	0.5747	0.1073	0.3643 to 0.7850	0.4899	>1.057	17.65 (3.799 to 43.43)	92.31 (63.97 to 99.81)	2.294
*N*-Glc MEP (**24**)	0.5249	0.1100	0.3092 to 0.7406	0.8180	>0.6104	35.29 (14.21 to 61.67)	92.31 (63.97 to 99.81)	4.588
*N*-Glc MEP (**25**)	0.7511	0.09376	0.5673 to 0.9350	0.02023	>1.053	38.46 (13.86 to 68.42)	82.35 (56.57 to 96.20)	2.179
*N*-Glc MEP (**26**)	0.6380	0.1041	0.4339 to 0.8421	0.2018	>1.029	30.77 (9.092 to 61.43)	82.35 (56.57 to 96.20)	1.744
CSF114(Glc) (**1**)	0.6516	0.1049	0.4458 to 0.8573	0.1610	>0.7575	47.06 (22.98 to 72.19)	92.31 (63.97 to 99.81)	6.118

## Supplementary Materials

The following are available online at https://www.mdpi.com/2076-3425/10/7/453/s1. Details of the synthetic procedures, analytical and immunochemical characterization of the synthetic antigenic probes are reported online. Table S1: Shortened CSF114(Glc)-sequences of peptides **2–8**., Table S2: Sequences and chemical data for the *N*-glucosylated (*N*-Glc) peptides **9–22**, Table S3: Analytical data of the Multiple *N*-glucosylated Peptide Epitopes (*N*-Glc MEPs) **23–26**, Table S4: Calculated half maximal inhibitory concentration (IC50). Calculated Log 1/IC50 with the Std errors and the IC50 with the Corresponding Confidence Interval (CI) for the shortened peptides versus CSF114(Glc), Table S5: Calculated Log 1/IC50 with the Std errors and the IC50 with the Corresponding Confidence Interval (CI) for the Peptides **9–22** and CSF114(Glc) (**1**), Figure S1: Received Operating Characteristic (ROC) analysis. ROC curve analysis of anti-CSF114(Glc) antibodies and anti-*N*-Glc MEPs **23–26** in Multiple Sclerosis versus controls determined by SP-ELISA, Figure S2: Competitive ELISA experiments with CSF114(Glc) and *N*-Glc MEP 24. Inhibition curves of anti-CSF114(Glc) antibodies with *N*-Glc MEP **24** compared with CSF114(Glc) in a competitive ELISA. The results are expressed as percentage of inhibition (ordinate axis) of three representative MS sera: MS1 (A), MS2 (B) and MS3 (C) versus the peptide concentrations (M) in logarithmical scale.
