# Supplementary material for: A Multiple N-Glucosylated Peptide Epitope Efficiently Detecting Antibodies in Multiple Sclerosis
Source: Brain Sci. 2020 Jul 15;10(7):453. doi: 10.3390/brainsci10070453 (PMC7408607; doi:10.3390/brainsci10070453)
Supplement: Supplementary file 1 [file brainsci-10-00453-s001.pdf]

Supplementary Materials

Synthetic Procedures

All Fmoc-protected amino acids, Fmoc-Wang resins, DIC (*N,N'* -Diisopropylcarbodiimide), Oxyma 2-(1Hbenzotriazol-1-yl)-1,1,3,3-tetramethyluronium hexafluorophosphaten(HBTU), and 1-[Bis(dimethylamino)methylene]-1H-1,2,3-triazolo[4,5-b] pyridinium 3-oxide hexafluorophosphate (HATU) were purchased were purchased from Iris Biotech GmbH (Marktredwitz, Germany). Peptide grade DMF was obtained from Scharlau (Barcelona, Spain). Triisopropyl silane (TIS) and 1,2-Ethanedithiol (EDT), DIPEA, diisopropyl ether (iPr_2O), diethylether (Et_2O) and Dry MeOH were purchased from Sigma Aldrich(Milano, Italy). HPLC-grade MeCN was purchased from Carlo Erba (Italy). Fmoc-*L*-Asn[β-D-Glc$(OAc)_4$]-OH were synthesized as previously described [*Paolini I, Nuti F, Pozo-Carrero MC, Barbetti F, Kolesinska B, Kaminski ZJ, Chelli M, Papini AM. A convenient microwave-assisted synthesis of N-glycosyl amino acids, Tetrahedron Letters 2007, 48(16), 2901-2904*].

General Procedure

General Procedure of Microwave-Assisted Solid Phase Synthesis

The β-turn glucopeptide CSF114(Glc) (**1**) was synthesized by microwave-assisted solid-phase synthesis (MW-SPPS) following the Fmoc/tBu strategy, using the Liberty Blue™ automated microwave peptide synthesizer (CEM Corporation, Matthews, NC, USA) following the protocol previously described [*Rizzolo F, Sabatino G, Chelli M, Rovero P, Papini AM. A convenient microwave-enhanced solid-phase synthesis of difficult peptide sequences: case study of Gramicidin A and CSF114(Glc). Int J Pept Res Ther 2007, 13(1-2), 203-208*]. Coupling of Fmoc-*L*-Asn[β-D-Glc$(OAc)_4$]-OH was performed using the adequately protected amino acids (2.5 eq), HATU as activator (2.5 eq), and DIPEA (3.5 eq) with 30W at 75°C in 300 sec.

General Procedure for in Batch SPPS on Manual Synthesizer

Peptides **2–26** were synthesized on a manual in batch synthesizer (PLS 4 × 4, Advanced ChemTech) using Teflon reactors (10 mL), on pre-loaded Wang resins and following the Fmoc/tBu SPPS procedure. The resin was swollen with DMF (1 mL/100 mg of resin) for 20 min before use. The synthesis was performed repeating the cycle described below for each amino acid: swelling: DMF (1 mL/100 mg of resin) for 5 min; Fmoc-deprotection: resin was washed twice with 20% piperidine in DMF (1 mL/100 mg of resin, one washing for 5 min and one for 20 min); resin-washings: DMF (3×5 min); the couplings were performed by Fmoc-amino acids (2.5 equiv), HBTU (2.5 equiv) and DIPEA (2.5 equiv) for 30 min. Each coupling was checked by Kaiser test and repeated if necessary; resin-washings: DMF (3 × 5 min) and DCM (3 × 5 min), Kaiser test. Uncertain peptide coupling results were checked by the ninhydrin test as described by Kaiser. [*Kaiser E, Colescott RL, Bossinger CD, Cook PI. Color test for detection of free terminal amino groups in the solid-phase synthesis of peptides, Anal Biochem 1970, 34(2), 595-598*] or micro-cleavages performed with a microwave apparatus CEM Discover™ single-mode MW reactor (CEM Corporation, Matthews, NC, USA).On completion of the synthesis the resin was washed with DCM and dried in *vacuo*.

General Cleavage Procedure

Cleavage from the resin and side-chain deprotections of peptides **2–8** (Table S1) were performed using a mixture of TFA/TIS/ethanedithiole/H_2O/ (93:2.5:2.5:2.5 v:v:v:v). The resin was treated for 2.5 h (1 mL/100 mg of resin) at r.t. Then it was filtered off and the solution was concentrated flushing with N_2. The peptides were precipitated from cold Et_2O, centrifuged and lyophilized.

Cleavage from the resin and side-chain deprotections of peptides **9–26** (Tables S2 and S3) were carried out with TFA/TIS/H_2O (95:2.5:2.5 v:v:v) for 3 h at r.t. The resin was filtered off and the

solution concentrated under N_2 flow. Crude products were precipitated with cold diisopropyl ether. The solid was centrifuged and washed with diisopropyl ether twice, and then lyophilized.

Synthesis of the N-Glc Peptides

N-Glc peptide epitopes **2–22** (Tables 1 and 2) were synthesized on a manual in batch synthesizer, starting from Rink Amide resin (250 mg, 0.63 mmol/g). *N*-Glc peptide epitopes **2–22** were acetylated on the N-terminal function with Ac_2O (10 equiv) and NMM (10 equiv) in DMF for 2 h. The peptides were cleaved and side-chains were deprotected as described in the general cleavage procedure. The deprotection of the hydroxyl functions of glucose was performed with MeONa/MeOH. The peptides were lyophilized and purified.

Synthesis of N-Glc Multiple Epitope Peptides (N-Glc MEPs)

N-Glc MEPs **23–26** (Figure 4) were synthesized, starting from $Fmoc_4$-Lys_2-Lys-β-Ala-Wang resin (100 mg, 0.2 mmol/g). Fmoc-*L*-Asn(β-GlcAc4)-OH (2.5 equiv), Fmoc-NH-(PEG)-COOH (19-atoms spacer) (N1-(9-Fluorenylmethoxycarbonyl)-1,13-diamino-4,7,10-trioxatridecan-succinamic acid) (2.5 equiv), Fmoc-NH-(PEG)-COOH (9-atoms spacer) (Fmoc-8-amino-3,6-dioxaoctanoic acid) (2.5 equiv) were coupled with HATU (2.5 equiv) and NMM (5 equiv) in DMF for 1 h. *N*-Glc MEPs **23–26** were acetylated on the N-terminal function with Ac_2O (10 equiv) and NMM (10 equiv) in DMF for 2 h. The peptides were cleaved and side-chains were deprotected as described in the general cleavage procedure. The deprotection of the hydroxyl functions of glucose was performed with MeONa/MeOH. The peptides were lyophilized and purified.

Purification step and characterization by RP-HPLC-MS

All peptides **1–26** were purified by semipreparative RP-HPLC on a Waters instrument (Separation Module 2695, detector diode array 2996) using a Phenomenex (Torrance, CA, USA) Jupiter column C18 (10 µm, 250 × 10 mm), at 4 mL/min with solvent system A (0.1% TFA in H_2O) and B (0.1% TFA in CH_3CN). The purity of the peptides was analysed by analytical HPLC using a Waters ACQUITY HPLC coupled to a single quadrupole ESI-MS (Waters 3100 Mass Detector) supplied with a BEH C18 (1.7 µm 2.1 × 50 mm) column at 35 °C, at 0.6 mL/min with solvent system A (0.1% TFA in H_2O) and B (0.1% TFA in CH3CN).

The peptides were purified by semi-preparative RP-HPLC and characterized by RP-HPLC and ESI-MS, obtaining a final purity ≥ 98%. Data were acquired and processed using MassLynx software (Waters, Milford, MA, USA). The analytical data are reported in details in Tables S1-S3.

Table S1. Shortened CSF114(Glc)-sequences of peptides **2**–**8**.

Peptides	HPLC (Rt, min)	ESI-MS (m/z) found (calcd)
[Asn^7(Glc)]CSF114 (1−18) (**2**)	14.58[a]	1145.1[d] (2288.1)
[Asn^7(Glc)]CSF114 (1−16) (**3**)	13.55[a]	1023.6[d] (2046.1)
[Asn^7(Glc)]CSF114 (1−14) (**4**)	13.81[a]	893.5[d] (1786.0)
[Asn^7(Glc)]CSF114(2−13) (**5**)	15.40[a]	807.4[d] (1613.9)
[Asn^7(Glc)]CSF114(4−11) (**6**)	6.51[a]	1100.8 (1101.2)
[Asn^7(Glc)]CSF114(5−10) (**7**)	3.85[b]	901.4 (902.9)
[Asn^7(Glc)]CSF114(6−9) (**8**)	3.77[c]	685.7 (686.7)

Analytical HPLC gradients at 1 mL min^{-1}; solvent system A: 0.1% TFA in H_2O, B: 0.1% TFA in CH_3CN; [a] 5–50% B in 20 min; [b] 0–30% B in 20 min; [c] 0–5% B in 20 min; [d] Detected as $[M+2H]^{2+}$.

Table S2. Sequences and chemical data for the *N*-glucosylated (*N*-Glc) peptides **9**–**22**.

Peptides	HPLC (Rt, min)	ESI-MS (m/z) Found [c] (calcd)
Ac-N(Glc)GS-NH_2 (**9**)	3.27 [a]	502.22 (502.45)
Ac-N(Glc)GT-NH_2 (**10**)	3.54 [a]	494.17 (494.48)
Ac-N(Glc)KS-NH_2 (**11**)	3.54 [a]	551.35 (551.26)

Ac-N(Glc)KT-NH_2 (**12**)	4.01 [a]	565.35 (565.28)
Ac-N(Glc)GH-NH_2 (**13**)	3.69 [a]	530.21 (530.52)
Ac-N(Glc)KH-NH_2 (**14**)	3.69 [a]	601.32 (601.29)
Ac-N(Glc)AT-NH_2 (**15**)	3.64 [a]	508.74 (508.51)
Ac-ERN(Glc)GS-NH_2 (**16**)	3.85[b]	765.48 (765.76)
Ac-ERN(Glc)GT-NH_2 (**17**)	3.80 [b]	779.12 (779.79)
Ac-ERN(Glc)KS-NH_2 (**18**)	3.97 [b]	695.02 (694.68)
Ac-ERN(Glc)KT-NH_2 (**19**)	3.88 [b]	837.13 (836.88)
Ac-ERN(Glc)GH-NH_2 (**20**)	3.87 [b]	814.93 (815.36)
Ac-ERN(Glc)KH-NH_2 (**21**)	3.76 [b]	808.96 (809.91)
Ac-KGN(Glc)AT-NH_2 (**22**)	3.84 [b]	887.01 (886.95)

Analytical HPLC gradients at 1 mL min^{-1}; solvent systems A: 0.1% TFA in H_2O, B: 0.1% TFA in CH_3CN; [a] 0-50% B in 15 min; [b] 0-30% B in 10 min; [c] Detected as $[M+H]^+$.

Table S3. Analytical data of the Multiple *N*-Glucosylated Peptide Epitopes (*N*-Glc MEPs) **23–26**.

***N*-Glc MEP**	**Analytical RP-HPLC gradients**	**HPLC (R_t, min)**	**ESI-MS (m/z) Found[a] (calcd)**
**23**	02-35% B 8 min	3.94	1016.42 (1017.98) $[M+2H]^{2+}$
**24**	05-40% B 8 min	3.96	1416.63 (1415.73) $[M+3H]^{3+}$
**25**	05-40% B 8 min	4.52	1219.93 (1219.26) $[M+4H]^{4+}$
**26**	03-25% B 8 min	4.01	1338.81 (1339.28) $[M+3H]^{3+}$

Analytical HPLC gradients at 0,6 mL/min; solvent systems: A: 0.1% TFA in H_2O, B: 0.1% TFA in CH_3CN; [a] Detected as [M+H]+.

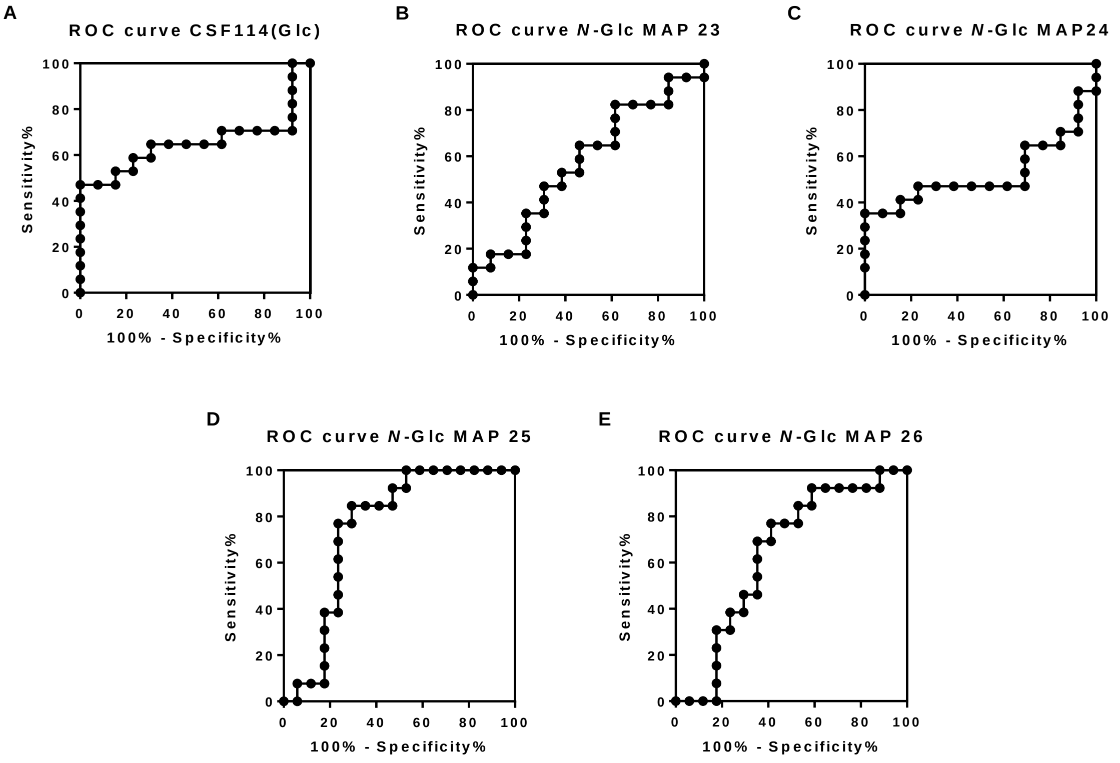

Figure S1. Received Operating Characteristic (ROC) analysis. ROC curve analysis of anti-CSF114(Glc) antibodies and anti-*N*-Glc MEPs **23–26** in Multiple Sclerosis versus controls determined by SP-ELISA.

Table S4. Calculated half maximal inhibitory concentration (IC50). Calculated Log 1/IC50 with the Std errors and the IC50 with the Corresponding Confidence Interval (CI) for the shortened peptides versus CSF114(Glc).

Peptide	Log1/IC50 ± Std Error	IC50 (μM) [CI 95%]
CSF114(Glc) (**1**)	6.14 ± 0.083	0.009 [0.006 ± 0.009]
[Asn^7(Glc)]CSF114 (1–18) (**2**)	7.1 ± 0.13	0.07 [0.027 ± 0.2]
[Asn^7(Glc)]CSF114 (1–16) (**3**)	6.8 ± 0.44	0.17 [0.007 ± 4.2]
[Asn^7(Glc)]CSF114 (1–14) (**4**)	7.9 ± 0.045	0.014 [0.009 ± 0.01]
[Asn^7(Glc)]CSF114 (2–13) (**5**)	7.5 ± 0.22	0.035 [0.006 ± 0.17]
[Asn^7(Glc)]CSF114(4–11) (**6**)	6.2 ± 0.29	0.56 [0.087 ± 3.62]
[Asn^7(Glc)]CSF114(5–10) (**7**)	5.4 ± 0.10	3.5 [1.84 ± 6.72]
[Asn^7(Glc)]CSF114(6–9) (**8**)	5.7 ± 0.25	2.2 [0.43 ± 10.2]

Table S5. Calculated Log 1/IC50 with the Std errors and the IC50 with the Corresponding Confidence Interval (CI) for the Peptides **9–22** and CSF114(Glc) (**1**).

Peptide	Log1/IC50 ± StdError	IC50(μM)[CI95%]
Ac-N(Glc)GS-NH_2(**9**)	5.137 ± 0.063	7.3 [4.8 ± 0.11]
Ac-N(Glc)GT-NH_2(**10**)	5.129 ± 0.1170	7.4 [3.5 ± 15.7]
Ac-N(Glc)KS-NH_2(**11**)	5.942 ± 0.1911	1.1 [33.7 ± 3.87]
Ac-N(Glc)KT-NH_2(**12**)	6.010 ± 0.06024	0.97 [66.5 ± 1.44]
Ac-N(Glc)GH-NH_2(**13**)	5.226 ± 0.04330	6.94 [4.5 ± 7.8]
Ac-N(Glc)KH-NH_2(**14**)	5.160 ± 0.01636	6.93 [6.24 ± 7.7]
Ac-N(Glc)AT-NH_2(**15**)	5.005 ± 0.05558	7 [6.93 ± 14.1]
Ac-ERN(Glc)GS-NH_2(**16**)	5.677 ± 0.1444	2.1 [0.84 ± 5.3]
Ac-ERN(Glc)GT-NH_2(**17**)	6.23 ± 0.14	0.58 [0.24 ± 1.43]
Ac-ERN(Glc)KS-NH_2(**18**)	5.59 ± 0.19	2.53 [0.75 ± 8.5]
Ac-ERN(Glc)KT-NH_2(**19**)	6.27 ± 0.022	0.54 [0.46 ± 0.62]
Ac-ERN(Glc)GH-NH_2(**20**)	5.95 ± 0.112	1.10 [0.54 ± 2.26]
Ac-ERN(Glc)KH-NH_2(**21**)	5.98 ± 0.1482	1.03 [0.4 ± 2.67]
Ac-KGN(Glc)AT-NH_2(**22**)	6.22 ± 0.07	0.60 [0.38 ± 0.94]
CSF114(Glc) (**1**)	6.46 ± 0.083	0.34 [0.12 ± 0.56]

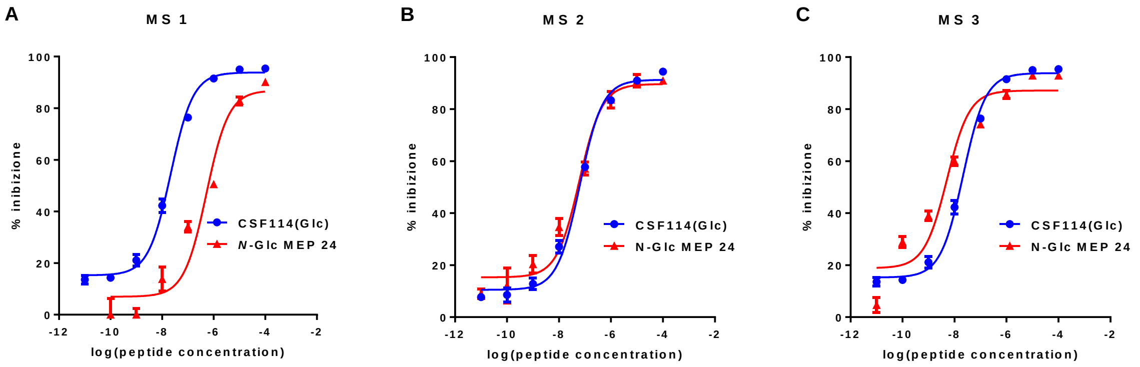

Figure S2. Competitive ELISA experiments with CSF114(Glc) and *N*-Glc MEP **24**. Inhibition curves of anti-CSF114(Glc) antibodies with *N*-Glc MEP **24** compared with CSF114(Glc) in a competitive ELISA. The results are expressed as percentage of inhibition (ordinate axis) of three representative MS sera: MS1 (A), MS2 (B) and MS3 (C) versus the peptide concentrations (M) in logarithmical scale.
